# “Tiptoeing Around the System”: Alternative Healthcare Navigation Among Gender Minorities in New Orleans

**DOI:** 10.1089/trgh.2018.0015

**Published:** 2018-07-01

**Authors:** Jennifer L. Glick, Katherine M. Andrinopoulos, Katherine P. Theall, Carl Kendall

**Affiliations:** ^1^ Department of Global Community Health and Behavioral Sciences, Tulane School of Public Health and Tropical Medicine, New Orleans, Louisiana.; ^2^ Department of Mental Health, Johns Hopkins Bloomberg School of Public Health, Baltimore, Maryland.

**Keywords:** access to care, alternative healthcare navigation strategy, gender minorities, health seeking behavior, transgender health

## Abstract

**Purpose:** Gender Minority (GM) individuals experience healthcare access barriers, including financial concerns and discrimination, which influence their health seeking behaviors. This study explores the alternative navigation strategies used by GM individuals to cope with these barriers and access care, both biomedical and complementary and alternative medicine (CAM).

**Methods:** In-depth interviews were conducted with GM individuals (*n*=18) and healthcare providers (*n*=5) identified through purposive sampling. Semistructured guides were used to elicit information about healthcare seeking strategies and experiences. Transcribed interview data were coded, sorted, and analyzed for key themes.

**Results:** Commonly discussed healthcare access barriers included the following: identifying a competent provider, costs and insurance obstacles, and anticipated discrimination. Respondents expressed a need for gender-affirming care within the biomedical system, and alternative navigation strategies to overcome access barriers, including travelling abroad for surgical procedures, ordering hormones online, and sharing with friends. Respondents discussed CAM principally related to emotional health, preferring CAM to biomedical offerings. Utilizing social networks to access all care modalities was common.

**Conclusions:** The healthcare-seeking behavior of GM individuals demonstrates great resilience. This population is committed to accessing gender-affirming care regardless of the associated risks of care outside of provider supervision. The healthcare community needs to eliminate access barriers and support harm reduction strategies. CAM for emotional health support and the role of social networks in accessing care resources should be better integrated into care for this population.

## Introduction

The current literature on transgender healthcare access and navigation demonstrates that gender minority (GM) adults report high rates of discrimination across multiple arenas, including healthcare, employment, and housing.^1–4^ Nationally, 23% of GM adults reported not seeing a doctor when needed in the past year due to fear of transgender related mistreatment.^5,6^ Our own research shows that GM individuals who experience discrimination in either health or nonhealth settings (such as transportation, civic, and commercial spaces) were more likely to delay accessing preventive care.^7^ Discrimination includes treatment refusal, verbal harassment, physical or sexual assault, or having to teach providers about transgender health.^5–7^

GM individuals, an umbrella category, including those identifying as transgender and gender nonconforming, report an array of healthcare access barriers related to (1) costs and insurance, (2) identifying culturally and medically competent providers, and (3) past experiences of discrimination when seeking healthcare.^8,9^ According to the United States Trans Survey (USTS), 33% of GM adults did not see a doctor when needed because they could not afford it.^5^ Many insurance plans do not cover gender-affirming care or gender-appropriate screenings (i.e., hormones for gender affirmation or pap smears for a transgender man or other transmasculine person who still retains a cervix). Twenty-five percent of USTS respondents experienced a problem with their insurance in the past year that was related to being transgender.^5,6^ Many GM people who sought coverage for transition-related surgery (55%) or hormones (25%) were denied in the past year.^5^ Further, another access barrier is the lack of competent medical providers, including having appropriate medical knowledge and interpersonal competence to treat GM patients.^10^

The healthcare challenges faced by GM individuals who have experienced access barriers to biomedical care may drive people to alternative navigation strategies, defined by the authors to include (1) nonnormative strategies to access biomedical care resources (medications and information) and (2) the use of complementary and alternative medicine (CAM). CAM is a group of diverse medical and healthcare systems, practices, and products that are not generally considered part of biomedicine; complementary medicine is used together with biomedicine and alternative medicine in place of it.^11^ Most of the research demonstrating a connection between discrimination and CAM use focuses on racial discrimination,^12–14^ although two studies focused on sexual minority women found that a lesbian sexual orientation and perceived discrimination in healthcare settings were both associated with CAM use.^15,16^

Many GM people desire hormones for gender affirming care. Illicit hormone use among GM people has been documented in multiple studies.^2,10,17–19^ A 2015 study in San Francisco found that 49.1% of transgender women reported taking hormones not prescribed by a clinician, and that the odds of this outcome increased for participants reporting verbal abuse due to their gender identity and presentation.^17^ Information on general health, gender affirming care, and hormone use is reported to come from a variety of sources outside of licensed medical practitioners. One study of trans feminine people showed that friends (57.7%) and the internet (25.4%) were the most common information sources after a general practitioner (73.2%).^10^ Other studies have shown that transgender people who obtained their hormones from physicians were more aware of side effects than those who used other sources to access hormones.^20^ This study accounts for barriers faced by GM individuals in accessing biomedical healthcare and explores the alternative navigation strategies they use to access needed healthcare, both within biomedical and CAM systems.

## Methods

This study utilized targeted ethnography, a rapid ethnographic method used in public health and social science inquiry,^21^ consisting of in-depth interviews with members of the GM community and healthcare providers over a period of 4 months in 2015. A semistructured field guide was developed for individual face-to-face interviews. Six pilot interviews were conducted to refine the guide before data collection. The final guide consisted of five sections, covering the following: (1) demographic information; (2) gender and sexual identities; (3) assessments of health and healthcare access; (4) past experiences of stigma and discrimination in various settings; and (5) personal experiences of illness and care-seeking. Example question prompts include (1) Have you faced more discrimination when seeking services for any particular type of health concern? How have you coped? (2) What types of complimentary or alternative health services exist in the New Orleans, Louisiana (NOLA) area? What sorts of self-care or home remedies do you or others utilize?

All transgender and gender nonconforming individuals of age 18 years and more living in NOLA, U.S. were eligible to participate. Flyers promoting the study were distributed online via relevant Facebook groups, the first author's personal Facebook page, and as an email attachment sent to individuals and organizations with connections to the GM community. People were encouraged to spread the word throughout the community. Hard copies of the flyer were distributed to providers at a community clinic offering gender competent care. The flyer encouraged potential participants to reach out to the study team. After completing the interview, participants were invited to refer others. While personal networks and organizational word of mouth sometimes overlapped, respondents primarily entered the study through the following engagement strategies: organization referrals (*n*=8), referred by participants or others who knew of the study (*n*=4), the first authors' personal network (*n*=4), and clinic referral (*n*=2). Of note, members of organizations serving the African American community as well as one white potential participant cited lack of financial incentive and research fatigue as reasons for nonparticipation. Healthcare providers with a focus on GM health were identified via GM references or the first author's professional network and were directly invited to participate.

The first author conducted, audiorecorded, and took field notes for all interviews, lasting approximately one hour. GM interviews continued until saturation was reached on key points. Upon completion of data collection, interviews were transcribed, and transcripts and notes were coded using NVIVO Version 11. Codes were developed *a priori* based on the research aims and *in vivo*, as the analysis process progressed. Data within the macro codes of “barriers to care,” “alternative navigation,” and “CAM” were primarily used to inform this analysis. These code reports were reviewed for emerging subthemes and data resorted accordingly. Memos were written throughout this process to facilitate synthesis of findings. Results are summarized throughout, and illustrative quotes are offered to underscore key points. The Tulane University Institutional Review Board approved the study. All participants provided written consent to participate.

## Results

The GM respondents (*n*=18) primarily identified as white (*n*=14) and ranged in age from 23 to 64. About half were originally from Louisiana, with half of those from New Orleans. The remaining respondents were from the United States and had been living in NOLA between 3 and 10 years. All respondents had previous experience using NOLA health services, including care for mental health issues (e.g., insomnia, anxiety), injuries, asthma, or HIV. Respondents' occupations varied, including artists, political activists, food service workers, and students. Most had health insurance, typically through a needs program or purchased by family or themselves via the Affordable Care Act (ACA) marketplace. To respect the self-determined gender identities of participants, Table 1 provides responses to the prompt, “How do you currently identify your gender?” along with a researcher ascribed simplified set of terms to be used when identifying respondents, assigned sex at birth, and sexual orientation data. For a thoughtful discussion regarding how and why researcher ascribed identifiers are used, please see Glick et al.^22^ The provider sample included three NOLA-based providers, an internist, endocrinologist, and pediatrician, and two providers outside of Louisiana, an urban LGBT focused clinic's Medical Director and a rural herbalist.

**Table 1.** **Information on Gender Minority Respondents' Gender Identity and Sexual Orientation (Providers Not Included)**

#	Gender identity^a^	Researcher ascribed gender label^b^	Assigned sex at birth	Sexual orientation
1	I go with genderqueer or gender fluid	Genderqueer person	F	Queer
2	Genderqueer. But with the qualifier of “raised as a girl,” generally shuffled into the “girl/woman” box by the world around me, which is certainly an influence	Genderqueer person	F	Queer
3	Trans male, so I identify as both male and transgender	Transgender male	F	Queer
4	I am a transgender female	Transgender female	M	Bisexual
5	That's a great question. Mostly I don't. Cause it doesn't bother me that much. That's not really true. But in some sense it feels, different. I dunno… Definitely gender non-conforming. I would identify as having a trans identity in that my experience of my own gender and how I would identify, which is probably non-binary and more masculine, more feminine identified male. I don't know, it's confusing. At one point I felt like I was failing to be gender queer in some ways and I was like, f^***^ it, I'm just not going to identify as that	Gender nonconforming person	F	Queer/dyke
6	My legal gender identity is female…Legal, physical, I'm female… I don't even use that term [transgender]; I don't think it technically applies anymore. I'm just a female person	Female (of transgender experience)	M	Bisexual/Asexual
7	Male	Male (of transgender experience)	F	Gay or queer
8	Trans, actually I identify as genderqueer. I think to be intelligible in more mainstream institutions I would use the Trans label. In terms of a true identification I identify as being queer. In terms of queer as a category which is critiquing other categorizations like LGBT	Genderqueer Person	F	Queer
9	Female…. I don't think I'd identify myself as genderqueer. I dunno. Maybe I would. I think that term means something that is deeper than what I feel. I feel like my gender is a different way of being a woman that is equally valid to all ways of being female or woman. I think some people who are GQ feel they are not a woman or female or in between or different…I don't conform to the standard of what women are supposed to look like. My sexuality is lesbian and my gender is queer, but gender queer implies something that maybe isn't what I am	Gender nonconforming woman	F	Lesbian
10	Me and Gonzo are of the same species, we're whatevers. I know that I am technically not female and, unless they can like, graft ovaries in me or something, I know that I'm never really gonna be that. But at the same time, I like fashion and I like looking and identifying as female	Transgender female	M	Asexual
11	It's kind of complicated. Usually, I say I'm transgendered because that's the easiest way to answer it. I mean, I definitely identify on the male spectrum but I live in-between male and female. It's a weird place. Not a lot of people choose to do it. It makes life complicated. It's kind of an evolving thing for me so I don't really feel like using a category. Generally, I say I'm transgendered, FTM. But I don't really fit into any category	Transmasculine person	F	Queer
12	I personally identify as, for data's sake, I identify as a trans woman of color. My person term that I coined for myself is FGD, female-gender dominant. So, I like to call myself that a lot	Trans woman	M	Queer
13	Genderqueer	Genderqueer person	M	Queer
14	Female	Female (of transgender experience)	M	Asexual
15	Female…“I've had the surgery, I am now a woman.”…I consider myself a woman but I'm also a trans woman I think	Female (of transgender experience)	M	Pansexual
16	Complicated. I identify on a transmasculine spectrum for sure, but it's a lot more fluid than that for me and I know that like, whatever the f^***^ passing means - I do sometimes and I don' t sometimes. And I don' t actually care to because I exist on the spectrum between male and female - much farther from female with no aspirations to be a man	Transmasculine person	F	Queer
17	Female	Female (of transgender experience)	M	Bisexual
18	I'm a guy. Straight up dude all the way	Man (of transgender experience)	F	Straight

^a^ In response to the prompt, “How do you currently identify your gender?”

^b^ Gender label provided for use in report.

### Biomedical healthcare access barriers

Commonly discussed healthcare access barriers included identifying a provider who is both transgender-friendly and transgender-knowledgeable, the cost of care and health insurance obstacles, and anticipated discrimination.

It's tough to find one, a doctor that understands what you're going through, cares what you're going through, and won't charge an arm and a leg to see you.

- Transgender Woman, 30, mixed race

I have so much anxiety from even thinking about [finding a healthcare provider] that it prevents me from even doing research myself and I don't even know what to do to start. Because healthcare is this black hole, it's like this ominous thing.

-Genderqueer Person, 35, white

Many participants, even those with insurance, discussed experiencing financial obstacles to accessing healthcare which, for example, made it difficult to obtain and adhere to prescription regimens. One respondent on an insurance plan with his estranged family discusses the challenge of meeting a yearly deductible.

I was on antidepressants and pretty strong anti-anxiety medication for a long time. It wasn't recommended that I go off but my health insurance got weird. All of a sudden the amount of money that I had to put out at the pharmacy to get my meds was almost $400, which is how much I pay for rent. And there weren't people that I can say, “Hey- Can you help me sort it out? I could pay you back eventually.” Instead I just tapered myself off of them, which is not really recommended.

-Transgender Man, 23, white

Nearly every respondent experienced discrimination in a healthcare setting; for example, refusal of services and verbal, physical, and sexual abuse. Some mentioned that discrimination in a medical location was particularly hurtful, differently so than in other spaces.

One of the other discriminating things about the doctor is that because they look at everything so clinical, they don't always make you feel valid. It's kind of like they break you down to your organs and your genitalia. It's like you're not a whole human being. Feeling broken down to just my sexual parts is really dehumanizing. I think that the discrimination I experience at the doctor, although it's more subtle and more explained away, hurts more because this is what you're saying about me medically.

-Trans Woman, 38, African American and Native American

Providers note the impact of discrimination on their patients as well.

I have witnessed slurs being hurled at my patients by the spouses of other patients. It's very difficult to get care where you're worried about your safety, but also when you're trying to be protective of your dignity. Shame is a horrible thing. Many of my patients have internalized the shame from the intolerance of others, which makes it [accessing healthcare] very hard.

- General practitioner and GM care provider

Many respondents have found alternative navigation strategies to access biomedical information and resources or are accessing CAM instead.

### Alternative navigation strategies: accessing biomedical gender-affirmation care

The majority of respondents focused their stories on gender-affirming healthcare and discussed travelling abroad for surgical procedures and ordering hormones and medication online via international pharmacies or from pharmacies in other states where they had previously established relationships. Some of this was cost driven and others used this strategy to avoid surveillance and having to interact with the biomedical care delivery system or establish new relationships with local providers.

Many respondents obtained hormones outside of standard biomedical procedures, for instance, by ordering them online without a prescription, sharing among friends, or stealing. Respondents educated themselves on dosage by talking to friends, reading online sources, and self-experimentation.

I was self-medicating with hormones for like a month and a half. I knew I needed help, that I couldn't do it [transition gender] on my own. The last thing I needed was for bad things to happen to me. Thankfully that didn't happen.

–Woman (of transgender experience), 48, white

I'll just hope that the next time I need a refill on my hormones I can figure it out or have a friend who's off of them right now who can call. That's another way, I have friends who still have prescriptions, who have refills and are not taking it right now or are on an off phase, you know? Or who can just call in to their doctor and be like, “Oh, I need a refill,”… I know tons of people that order them from Canada. It's a pretty regular thing to do because it's so hard to jump through all the hoops of getting them here.

-Transgender Male, 23, white

Respondents also discussed covert body modification with illicit silicone as a health issue for GM people and the importance of identifying safe providers outside of the biomedical sphere.

There's tons of underground body modification stuff besides hormones, like silicone injections. We've seen a lot of that in the past few years. A lot of times it's not actually silicone, it's concerning. There've been a few trans people who have pretty major health concerns because of street silicone injections.

-Transgender Male, 23, white

I had some silicone procedures here in the last 5 years. It was all on the black market…[Interviewer: what was that like?] It's different. It depends on the individual that's administering. You gotta find someone who likes you, knows what they're doing, and has the proper product. You have to test the product. If you don't know what the product is then you shouldn't even be bothering. If you don't know what the real deal is, leave it alone.

- Transgender Woman, age undisclosed, African American

A few respondents discussed telling care providers what they thought the provider wanted to hear or translating their actual experiences into language and scenarios that they thought would be more “legible” to the providers. This was particularly the case around issues of gender and sexuality.

When I go to a gynecologist, I feel like I'm always translating the questions they ask for my experience of gender and sexuality. Maybe you would get more info that was useful for what you wanted to know about my sexual health and I would get more info that was useful about my sexual health if we could talk more openly about what my gender is and actually the gender of the people I am sleeping with, or what gender roles you assume I have during sex. For example, I mostly identify sexually as a top and I am usually the person doing the f***ing, aspects of that are related to my sexual and gender identity. A gynecologist asks who I am sleeping with… men. But I'm sleeping with a trans man. What you see is a penis in my vagina when really what's happening is I'm doing the penetrating to the other person who is male identified… I haven't had experiences where I feel hurt but the language the provider uses has been irrelevant to my actual experience. And I mostly haven't been in experiences where I've felt it was worth it to explain. If I have sexual health questions, I'll ask friends who are sex educators or look on the Internet. And if I have a STD scare I might go to the provider and make up a scenario that seems to me to be a relevant scenario that would be legible to the provider with the same risk factors.

-Gender Non-Conforming Person, 32, white

### Alternative navigation strategies: CAM

About half of respondents discussed the use of CAM themselves or among peers. Modalities mentioned included herbs, acupuncture, yoga, meditation, reiki, bodywork (including massage therapy and rolfing), supplements, energy healing, and chiropractic. Respondents discussed alternative therapies for nongender-related health issues and expressed a belief in various forms of CAM to support overall holistic wellbeing while going through hormone therapy. Overwhelmingly, CAM resources were discussed as they relate to mental and emotional support that may or may not be related to gender affirmation therapy. Respondents discussed using massage and various movement or embodiment techniques both as complementary and alternative to talk therapy and medication. Respondents raised the importance of bodywork and embodiment modalities to facilitate better relationships to one's body, but that it could also be triggering for this population. People also discussed using herbs to manage emotional wellness, spiritual practices such as tarot and altar building, and participating in various group support activities, which they discussed as complementary or alternative.

I actually did a lot of therapy, different kinds of bodywork, and, I don't even know what you would call this [group empowerment course] …It was interesting, I got some stuff out of it. I was on anti-depressants for a time. Most of them made me sick but there was one I was on for almost a year that was helpful when I was about 30. I did a large amount of rolfing when I was in my early 30's and that lifetime of depression and stress was all inside of my body and rolfing just let it out…. for the last 5 years I've been doing 12 step work and I've gotten a huge amount out of that.

- Trans Masculine Person, 50, white

While many respondents discussed the inadequacy of biomedical care in NOLA, the network of alternative care providers was perceived as strong. Particularly among the “punk/queer^†^” community, providers were often friends, which alleviated some of the access barriers. Respondents outside that community discussed familiarity with alternative therapies but were less interested in using it themselves.

Informal [care networks] is pretty strong… maybe because people felt disenchanted with, or that it was inaccessible, or never had experience within allopathic systems. There is a lot of opportunity for herbalists, or offering alternative mental health services— embodiment, energy work— that exists down here.

-Gender Non-Conforming Person, 32, white

Respondents cited financial barriers to accessing CAM, especially because many CAM therapists are not supported by the health insurance system, although others discussed the availability of services through their community and social networks.

Most trans persons I know go to Western herbalists. Partially, I think it has to do with cost of care. Acupuncture is great but even if you're doing a community model, it's still expensive for a lot of people. Ayurvedic medicine is really good at treating you individually— Chinese medicine as well, more so than Western medicine. Anything that treats people constitutionally instead of just disease-based, is gonna be beneficial to trans folks. Affordability is the problem. People charge a lot of money.

-Herbalist and Gender Affirming Care provider

If I had more money I would see them [CAM providers] all the time. I would also see a [mental health] therapist. A therapist, a rolfer, a massage therapist… For trans people in particular it's really important to have someone touching your body.

- Genderqueer Person, 44, white

### Alternative navigation strategies: use of social networks

Respondents discussed utilizing social networks to access biomedical care and CAM-mentioning friends or family members who write them prescriptions, make house calls, and offer advice.

My partner had really bad asthma so she got this friend to call in a prescription. It's partly out of convenience, but it's also like, I don't want to go to a doctor's office. So I think people use informal networks if they have access to that… I f***ed up my knee and I called a friend of mine that is a queer physical therapist. She came by and I paid her like, $20. The things she did really worked.

-Gender Non-Conforming Woman, 31, white

While having the social capital to access providers outside of normal channels is a benefit, it can come with costs. One respondent expressed how the overlap of providing health services has put a burden on their friendship, but both people know that this is the best option to keep the patient in some sort of care.

I'm essentially dealing with people in a department that has no knowledge, understanding, or focus on trans healthcare. I am just like sort of tiptoeing around the system because I know someone. They're having to do all of this extra legwork which is annoying for them. So basically it's like people doing me favors.

-Trans-Masculine Person, 31, Native American

One respondent shared a story of seeking mental health support from a herbalist friend and how the social connection supported their ongoing treatment.

It was a huge thing for me, trusting someone from my community who I could open up to. She responded cool, calm, and collected… and then checked in. And then the fact that she's in my social world and would see me and be like, “How's it going?” That connection really helped me out of some stuff. It was nice because I don't think that a lot of our problems with health can be solved by our friends and people in our social circle but I think that when the right circumstance like that comes up, it can do way more than going to visit somebody in an office that you don't know.

- Genderqueer Person, 35, White

## Discussion

In this study, we documented a range of alternative healthcare navigation strategies used by GM individuals residing in NOLA. Participant narratives illustrate a desire for increased access to higher quality biomedical care, unobstructed by cost barriers and health insurance obstacles, wherein they are treated with dignity, able to openly discuss health issues, and can obtain accurate and technically competent care.

GM people face a wide array of gender-based discrimination and healthcare access barriers. While previously not documented for this understudied population in NOLA, this finding resonates with multiple studies of healthcare access across settings.^23^ Participants expressed a need for gender-affirming care within the biomedical system and alternative navigation strategies because of aforementioned obstacles. Conversely, participants reported the use and preference of CAM over biomedical care for emotional health. In both cases, respondents in this study called upon social networks to achieve access to care. The health seeking behavior of GM individuals in NOLA demonstrates great resilience and ingenuity in the alternative navigation strategies utilized. Notwithstanding, there is a clear need to remove structural barriers to care, including cost, insurance coverage, and increased access to providers who are knowledgeable and nondiscriminating for gender-affirming care.

Accessing biomedical resources without the care of a biomedical care provider means that people may not be using research-informed dosage guidelines or receiving recommended monitoring.^24^ Our study corroborates findings^18^ suggesting that negative provider experiences play a role in deciding to access illicit hormones. Efforts to improve access and quality of services for GM patients (i.e., antidiscrimination policies and cultural competency trainings for staff and providers) must be made to reduce the fear and mistrust of biomedical healthcare providers and promote accessing these resources under recommended care guidelines.

In the case of gender-affirming healthcare, harm-reduction strategies could be used to mitigate risks associated with the alternative navigation strategies. Examples of existing harm-reduction strategies, which can be supported and enhanced, include resource guides of local providers competent in GM health^25,26^; zines, pamphlets, and other forms of information sharing related to healthcare outside of biomedical settings^27^; needle exchange programs for safe hormone injection; and funding strategies to support biomedical costs and legal name changes. Of note, the vast majority of this work is being done by members of the community and further underscores the role of social support. Currently, there are both community organization funds^28^ for these needs as well as crowdfunding efforts.^29,30^ Harm-reduction approaches should be supported concurrent with changes to the biomedical system, not in lieu of them.

Participants presented productive CAM solutions for emotional health, similar to strategies used by many in the general population. A U.S. nationally representative survey reported that just over half of those with anxiety attacks and/or severe depression reported using CAM to treat these conditions.^31^ Models for providing integrative care exist in multiple contexts. For example, the ACA provides coverage for acupuncture in five states, not including Louisiana.^32^ A Washington state law mandates that insurance companies extend coverage to all categories of licensed providers,^33^ including CAM, and organizations exist to fill financial access gaps by offering holistic and integrative healthcare for people experiencing homelessness and extreme poverty.^34^ Furthermore, approximately 30 academic health centers across the United States and Canada currently deliver multidisciplinary integrative medicine, a combination of conventional and complementary medical services in one location.^35^

The use of social networks emerged as a key theme in navigation strategies. Alternative family structures and strong community supports are frequent characteristics of queer communities,^36,37^ and particularly characteristic of the “punk/queer” community in NOLA, which is anecdotally associated with a wide national network of social connection. The data showed more use of social networks to access CAM for those within this community, suggesting that GM individuals outside this smaller subset may not have access to this resource. However, the larger social marginalization and intersectional discrimination experienced, as evidenced by our study and many others,^5,38^ indicate that not all GM people are linked into a strong resourced social network and factors such as racism, classism, and misogyny within and outside the GM community may impact connections. For example, racially disadvantaged communities may have a different constellation of resources than those with more privilege. So while black trans women may be socially networked and support each other, there is less financial capital in that community to draw on when offering that support. The intersection of these oppressive systems has exponential impacts; social identities and inequality are interdependent for marginalized groups.^39^

While overall seen as a strength, overreliance on social network structures to access quality healthcare may have unintended consequences. Not all GM individuals have access to care through their social networks or want to use personal relationships to resolve healthcare issues, issues of confidentiality arise, among others. In this study, there were mixed perspectives on the level of comfort participants had using their social networks for healthcare. From a larger ethical perspective, overrelying on social networks can mask the need for larger structural change. By meeting one's needs through social capital, the gaps in the formal system appear less crucial. GM communities should not be expected to solve wider system problems by only looking within.

It is the authors' hopes that these findings can inform shifts in policy and practice as discussed above, or at minimum, a more thoughtful approach to engaging GM patients in healthcare. Research next steps include further examination of factors which influence healthcare seeking behavior in GM populations, including intersectional analysis for relevant disparities between subgroups of this population considering race/ethnicity, income, ability, nationality, and so on. Further research into the health impacts of illicit gender affirmation hormone use is necessary. Finally, research driven and evaluated intervention development to address existing barriers to standard biomedical care access and build on existing alternative navigation strategies can promote health in this population.

The current study has several strengths. First, research related to GM populations is limited, particularly in the Deep South, where there is a lack of healthcare access overall.^40^ Second, the sample includes diversity of gender experiences, including trans feminine, trans masculine, and gender nonconforming people, and people at different stages of and with different desires for transition. Finally, the qualitative approach offers nuanced understandings and is useful in exploring these dynamics in other GM populations and groups facing rampant discrimination.

One limitation was the relatively small sample size. While there was diversity in gender identity and age, limited racial, class, and income diversity may have concealed further discrimination, disparities, and access strategies. The GM community is known to face educational and employment discrimination, which impacts insurance access and overall income, known influences on healthcare seeking behavior.^5,38^ Some of the black individuals and one white individual interested in participating cited lack of financial incentive and research fatigue as reasons for nonparticipation, which is in alignment with literature on the obstacles of engaging this community in research.^22^ These points notwithstanding, saturation was reached on an array of findings, such as types of discrimination experiences, feelings about healthcare providers and access, health-seeking behavior, and alternative healthcare navigation strategies.

## Abbreviations Used

ACA: Affordable Care Act
CAM: complementary and alternative medicine
GM: gender minority
NOLA: New Orleans, Louisiana
USTS: United States Trans Survey

## Notes

^†^ Upon analysis of the data, an informal social subgroup emerged among 8 of the 18 respondents. While not queried about in the interviews, the first authors' own affiliation with this group provided relevant insight to identify affiliation and loosely describe it. This “queer/punk” group is a network of people with similar, yet not identical politics and philosophies around radical social change. A DIY (Do it yourself) ethic is prominent, in alignment with the philosophies of early punk music, and a rejection of social categories and oppressive forces, in alignment with the tenets of queer theory and intersectional feminism.

## References

[1] Lombardi EL, Wilchins RA, Priesing D, Malouf D Gender violence: transgender experiences with violence and discrimination. J Homosex. 2002;42:89–101 10.1300/j082v42n01_05 11991568

[2] Clements-Nolle K, Marx R, Guzman R, Katz M HIV prevalence, risk behaviors, health care use, and mental health status of transgender persons: implications for public health intervention. Am J Public Health. 2001;91:91 5 10.2105/ajph.91.6.915 PMC1446468 11392934

[3] Clements-Nolle K, Marx R, Katz M Attempted suicide among transgender persons: the influence of gender-based discrimination and victimization. J Homosex. 2006;51:53–69 17135115 10.1300/J082v51n03_04

[4] Bradford J, Reisner SL, Honnold JA, Xavier J Experiences of transgender-related discrimination and implications for health: results from the Virginia Transgender Health Initiative Study. Am J Public Health. 2013;103:1820–1829 23153142 10.2105/AJPH.2012.300796 PMC3780721

[5] James SE, Herman JL, Rankin S, et al. The Report of the 2015 US Transgender Survey. Washington, DC: National Center for Transgender Equality, 2016

[6] 2015 U.S. Transgender Survey: Louisiana State Report. Washington, DC: National Center for Transgender Equality, 2017 10 2017 Report No

[7] Glick JL, Theall KP, Andrinopoulos KM, Kendall C The role of discrimination in care postponement among trans-feminine individuals in the US National Transgender Discrimination Survey. LGBT Health. 2018;5:171–179 29589995 10.1089/lgbt.2017.0093 PMC6425922

[8] Gonzales G, Henning-Smith C Barriers to care among transgender and gender nonconforming adults. Milbank Q. 2017;95:726–748 29226450 10.1111/1468-0009.12297 PMC5723709

[9] Safer JD, Coleman E, Feldman J, et al. Barriers to healthcare for transgender individuals. Curr Opin Endocrinol Diabetes Obes. 2016;23:168–171 26910276 10.1097/MED.0000000000000227 PMC4802845

[10] Sanchez NF, Sanchez JP, Danoff A Health care utilization, barriers to care, and hormone usage among male-to-female transgender persons in New York City. Am J Public Health. 2009;99:713–719 19150911 10.2105/AJPH.2007.132035 PMC2661470

[11] Health UDo, Services H The use of complementary and alternative medicine in the United States. National Center for Complementary and Alternative Medicine. Available from: http://nccam nih gov/news/camstats/2007/camsurvey_fs1 htm Updated February. 2013;20

[12] Bazargan M, Norris K, Bazargan-Hejazi S, et al. Alternative healthcare use in the under-served population. Ethn Dis. 2004;15:531–539 16259473

[13] Choi NG, Kim J Utilization of complementary and alternative medicines for mental health problems among Asian Americans. Commun Ment Health J. 2010;46:570–578 10.1007/s10597-010-9322-4 20490674

[14] Shippee TP, Schafer MH, Ferraro KF Beyond the barriers: racial discrimination and use of complementary and alternative medicine among Black Americans. Soc Sci Med. 2012;74:1155–1162 22386637 10.1016/j.socscimed.2012.01.003 PMC3341177

[15] Matthews AK, Hughes TL, Osterman GP, Kodl MM Complementary medicine practices in a community-based sample of lesbian and heterosexual women. Health Care Women Int. 2005;26:430–447 16020008 10.1080/07399330590933962

[16] Smith HA, Matthews A, Markovic N, et al. A comparative study of complementary and alternative medicine use among heterosexually and lesbian identified women: data from the ESTHER Project (Pittsburgh, PA, 2003–2006). J Altern Complement Med. 2010;16:1161–1170 21058883 10.1089/acm.2009.0444 PMC3111143

[17] De Haan G, Santos G-M, Arayasirikul S, Raymond HF Non-prescribed hormone use and barriers to care for transgender women in San Francisco. LGBT Health. 2015;2:313–323 26788772 10.1089/lgbt.2014.0128

[18] Rotondi NK, Bauer GR, Scanlon K, et al. Nonprescribed hormone use and self-performed surgeries: “do-it-yourself” transitions in transgender communities in Ontario, Canada. Am J Public Health. 2013;103:1830–1836 23948009 10.2105/AJPH.2013.301348 PMC3780733

[19] Xavier J, Honnold JA, Bradford JB The Health, Health-related Needs and Life Course Experiences of Transgender Virginians. Richmond, VA: Virginia Department of Health, 2007

[20] Mepham N, Bouman WP, Arcelus J, et al. People with gender dysphoria who self-prescribe cross-sex hormones: prevalence, sources, and side effects knowledge. J Sex Med. 2014;11:2995–3001 25213018 10.1111/jsm.12691

[21] Wainberg ML, Alfredo González M, McKinnon K, et al. Targeted ethnography as a critical step to inform cultural adaptations of HIV prevention interventions for adults with severe mental illness. Soc Sci Med. 2007;65:296–308 17475382 10.1016/j.socscimed.2007.03.020 PMC2175036

[22] Glick JL, Theall K, Andrinopoulos K, Kendall C For data's sake: dilemmas in the measurement of gender minorities. Cult Health Sex. 2018:1–16 10.1080/13691058.2018.1437220 PMC6526522 29533145

[23] Reisner SL, Poteat T, Keatley J, et al. Global health burden and needs of transgender populations: a review. Lancet. 2016;388:412–436 27323919 10.1016/S0140-6736(16)00684-X PMC7035595

[24] Coleman E, Bockting W, Botzer M, et al. Standards of care for the health of transsexual, transgender, and gender-nonconforming people, version 7. Int J Transgenderism. 2012;13:165–232

[25] New Orleans Community Resource Guide For Resistance and Renewal. New Orleans, LA: Center for Ethical Living and Social Justice Renewal (CELSJR), 2018

[26] Advocates LT Louisiana Trans Advocates- Provider List. New Orleans, LA: Louisiana Trans Advocates, 2017

[27] BreakOUT. Guide to Harm Reduction in Injecting [In Spanish]. New Orleans, LA: BreakOUT, 2017

[28] Donna Jean Loy Memorial Assistance Fund: Louisiana Trans Advocates; 2018 [cited 2018 3 26, 2018]. Available from: www.latransadvocates.org/donna-jean-loy-assistance-fund

[29] Fritz N, Gonzales A Privacy at the Margins| Not the Normal Trans Story: negotiating Trans Narratives While Crowdfunding at the Margins. Int J Commun. 2018;12:2 0

[30] Farnel M Kickstarting trans*: the crowdfunding of gender/sexual reassignment surgeries. New Media Soc. 2015;17:215–230

[31] Kessler RC, Soukup J, Davis RB, et al. The use of complementary and alternative therapies to treat anxiety and depression in the United States. Am J Psychiatry. 2001;158:289–294 11156813 10.1176/appi.ajp.158.2.289

[32] Fan AY “Obamacare” covers fifty-four million Americans for acupuncture as Essential Healthcare Benefit. J Integr Med. 2014;12:390–393 25074889 10.1016/S2095-4964(14)60035-2

[33] Watts CA, Lafferty WE, Baden AC The effect of mandating complementary and alternative medicine services on insurance benefits in Washington State. J Altern Complement Med. 2004;10:1001–1008 15673994 10.1089/acm.2004.10.1001

[34] Outreach IC Integrative Care Outreach 2018 [cited 2018 3 26, 2018]. Available from: https://integrativecareoutreach.org

[35] Eisenberg DM, Kaptchuk T, Post DE, et al. Establishing an integrative medicine program within an academic health center: essential considerations. Acad Med 2016;91:122 3 10.1097/ACM.0000000000001173 PMC5007186 27028029

[36] Dewaele A, Cox N, Van den Berghe W, Vincke J Families of choice? Exploring the supportive networks of lesbians, gay men, and bisexuals. J Appl Soc Psychol. 2011;41:312–331

[37] Arnold EA, Sterrett-Hong E, Jonas A, Pollack LM Social networks and social support among ball-attending African American men who have sex with men and transgender women are associated with HIV-related outcomes. Global Public Health. 2018;13:144–158 27169632 10.1080/17441692.2016.1180702 PMC5106335

[38] Grant JM, Mottet L, Tanis JE, et al. Injustice at Every Turn: A Report of the National Transgender Discrimination Survey. Washington: National Center for Transgender Equality and National Gay and Lesbian Task Force, 2011

[39] Bowleg L When Black+ lesbian+ woman≠ Black lesbian woman: the methodological challenges of qualitative and quantitative intersectionality research. Sex Roles. 2008;59:312–325

[40] Okoro CA Surveillance for health care access and health services use, adults aged 18–64 years—behavioral risk factor surveillance system, United States, 2014. MMWR Surveill Summ. 2017;66:1–149 10.15585/mmwr.ss6607a1 PMC5829627 28231239

